# Crystal structure of 4,4′-di­nitro-[1,1′-biphen­yl]-2-amine

**DOI:** 10.1107/S205698901700408X

**Published:** 2017-03-21

**Authors:** Md. Serajul Haque Faizi, Ashanul Haque, Necmi Dege, Syed Imran Hasan, Mustafa Dege, Valentina A. Kalibabchuk

**Affiliations:** a Department of Chemistry, College of Science, Sultan Qaboos University, PO Box 36 Al-Khod 123, Muscat, Sultanate of , Oman; b Ondokuz Mayıs University, Arts and Sciences Faculty, Department of Physics, 55139 Samsun, Turkey; c Spraying Systems Company Turkey, Esentepe Mah. Kore Şehitleri Cad. Kaya Aldoğan Sok., Serhan apt. No. 3 Daire:3 Şişli İstanbul, Turkey; d Department of General Chemistry, O. O. Bohomolets National Medical University, Shevchenko Blvd. 13, 01601 Kiev, Ukraine

**Keywords:** crystal structure, di­nitro, biphen­yl, amine, biphenyl derivatives, hydrogen bonding

## Abstract

In the title biphenyl derivative, C_12_H_9_N_3_O_4_, the dihedral angle between the benzene rings is 52.84 (10)°. The nitro group attached to the benzene ring is inclined to the ring by 4.03 (2)°, while the nitro group attached to the amino-substituted benzene ring is inclined to the ring by 8.84 (2)°. In the crystal, mol­ecules are linked by two pairs of N—H⋯O hydrogen bonds, forming chains propagating along [101]. Within the chains, these N—H⋯O hydrogen bonds result in the formation of *R*_2_^2^(20) and *R*_2_^2^(14) ring motifs. The latter ring motif is reinforced by a pair of C—H⋯O hydrogen bonds, enclosing *R*_2_^1^(6) ring motifs. The chains are linked by a second C—H⋯O hydrogen bond, forming a three-dimensional supra­molecular structure.

## Abstract 2

In the title biphenyl derivative, the dihedral angle between the benzene rings is 52.84 (10)°. In the crystal, mol­ecules are linked by two pairs of N—H⋯O hydrogen bonds, forming chains propagating along [101].

## Chemical context   

Biphenyl and its derivatives have been shown to play an important role in fighting cancer and arteriosclerosis in humans (Umeda *et al.*, 2005 ▸). The dihedral angle between the phenyl rings of biphenyl derivatives is associated with their affinity for cellular target mol­ecules and, therefore, can correlate with their toxicity. The parent compound, biphenyl, adopts a planar conformation in the solid state with a dihedral angle of 0° (Trotter, 1961 ▸). The calculated dihedral angle for biphenyl derivatives without *ortho* substituents is *ca* 41° (Shaikh *et al.*, 2008 ▸). Deviations from the energetically most favourable conformation are most likely the result of crystal packing effects, which allow such compounds to adopt an energetically favorable conformation in the solid state by maximizing the lattice energy. Many research groups have calculated the inter-ring torsion angle of biphenyl in the solid state (Brock, 1980 ▸; Brock & Minton, 1989 ▸; Bastiansen & Samdal, 1985 ▸), and in the gas phase (Bastiansen & Traetteberg, 1962 ▸). We report here a detailed description of the mol­ecular structure and supra­molecular features of the title biphenyl derivative, 4,4′-di­nitro-[1,1′-biphen­yl]-2-amine, (I).
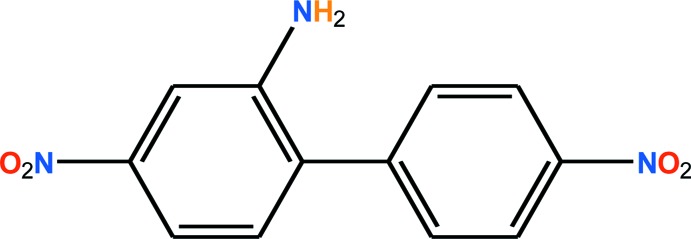


## Structural commentary   

**Figure 1:**
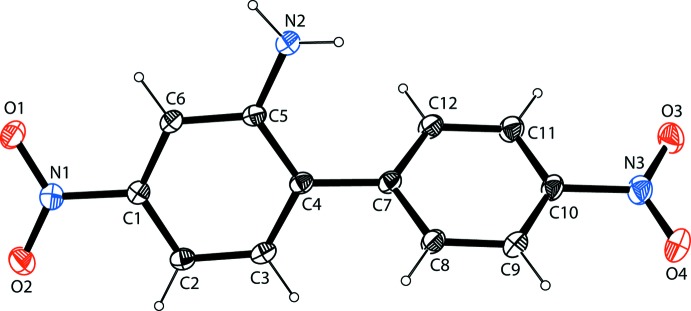
The mol­ecular structure of the title compound, with the atom labelling. Displacement ellipsoids are drawn at the 40% probability level.

The mol­ecular structure of the title compound (I), is illus­trated in Fig. 1 ▸. The dihedral angle between the two rings of the biphenyl unit is 52.84 (10)°. The nitro group (N3/O3/O4) is inclined to the benzene ring (C7–C12) to which it is attached by 4.03 (2)°. The nitro group (N1/O1/O2) is inclined to the amino-substituted benzene ring (C1–C6), to which it is attached, by 8.84 (2)°. The amino N atom, N2, lies in the plane of the C1–C6 benzene ring, and the N2—C5 bond length of 1.375 (3) Å clearly indicates a single bond. The C1—N1 distance of 1.466 (3) Å is slightly less than the C10—N3 bond distance of 1.477 (3) Å, which indicates that the 2-amino group containing a benzene ring (C1–C6) is more conjugated with the nitro group (N1/O1/O2) than is the other nitro group (N3/O3/O4) with respect to the C7–C12 benzene ring. The bond length of the C4—C7 bridge is 1.482 (3) Å, which indicates a single bond, and is similar to the same bond length of 1.494 (2) Å reported for dimethyl 2,2′-di­nitro­biphenyl-4,4′-di­carboxyl­ate (Lehane *et al.*, 2014 ▸), and *ca* 1.493 Å observed in 2,2′-di­nitro­biphenyl (Sekine *et al.*, 1994 ▸).

## Supra­molecular features   

**Figure 2:**
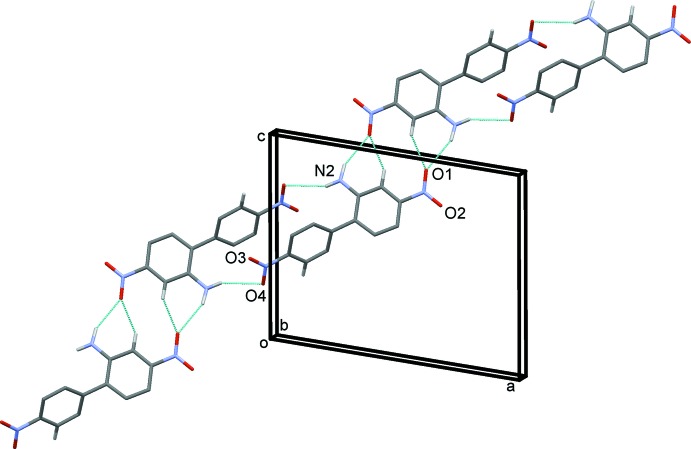
A view of the N—H⋯O and C—H⋯O hydrogen bonds (dashed lines; see Table 1 ▸), in the crystal of (I), forming chains that propagate along [101].

**Figure 3:**
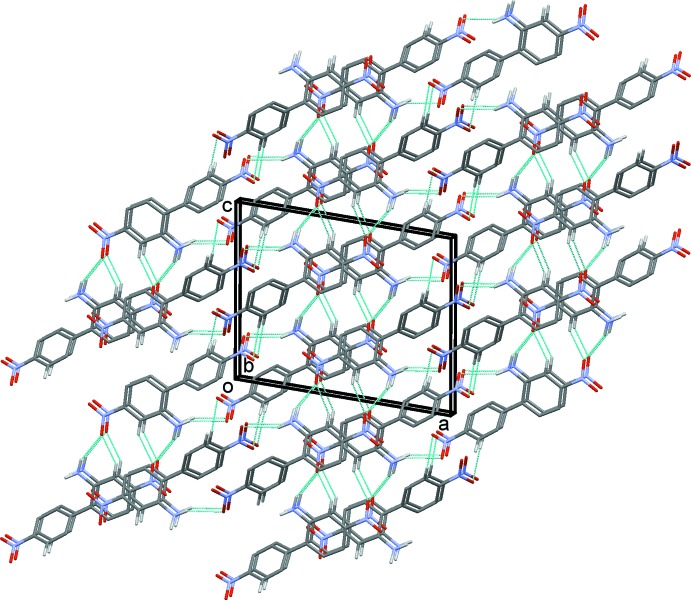
A view along the *b* axis of the crystal packing of (I). Hydrogen bonds are shown as dashed lines (see Table 1 ▸) and, for clarity, only H atoms H2*A*, H2*B*, H6 and H9 have been included.

In the crystal, mol­ecules are linked by two pairs of N—H⋯O hydrogen bonds, forming chains propagating along the [101] direction. Within the chains, these N—H⋯O hydrogen bonds result in the formation of 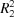
(20) and 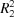
(14) ring motifs (Table 1 ▸ and Fig. 2 ▸). The latter ring motif is reinforced by a pair of C—H⋯O hydrogen bonds, enclosing 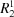
(6) ring motifs (Table 1 ▸ and Fig. 2 ▸). The chains are linked by a second C—H⋯O hydrogen bond (Table 1 ▸), forming a three-dimensional supra­molecular structure, as illustrated in Figs. 3 ▸ and 4 ▸.

## Database survey   

A search of the Cambridge Structural Database (CSD, Version 5.38, update February 2017; Groom *et al.*, 2016 ▸) revealed the structure of two similar compounds *viz* 4′-nitro-2-bi­phenyl­amine (II) (CSD refcode DIWFEU; Sutherland & Ali-Adib, 1986 ▸) and 4,4′-di­nitro­biphenyl (III) (DNTDPH; Boonstra, 1963 ▸). In (II), the benzene rings are inclined to one another by 54.64 (6)°, compared to *ca* 32.91° in (III), and to 52.84 (2)° in the title compound (I). In (II), the nitro group is inclined to the benzene ring to which it is attached by 7.08 (6)°, compared to *ca* 3.55 and 10.14° in (III) and 8.3 (2)° in the title compound (I).

## Synthesis and crystallization   

The title compound (I), was prepared by a literature procedure (Ol’khovik *et al.*, 2008 ▸). Orange prismatic crystals, suitable for single-crystal X-ray analysis, were grown by slow evaporation of a solution in ethanol.

## Refinement   

Crystal data, data collection and structure refinement details are summarized in Table 2 ▸. The N-bound H atoms were located in a difference Fourier map and refined with *U*_iso_(H) = 1.2*U*_eq_(N). The C-bound H atoms were included in calculated positions and refined as riding: C—H = 0.93–0.96 Å with *U*_iso_(H) = 1.2*U*_eq_(C).

## Supplementary Material

Crystal structure: contains datablock(s) I, Global. DOI: 10.1107/S205698901700408X/su5355sup1.cif

Structure factors: contains datablock(s) I. DOI: 10.1107/S205698901700408X/su5355Isup2.hkl

Supporting information file. DOI: 10.1107/S205698901700408X/su5355Isup3.cml

CCDC reference: 1537734

Additional supporting information:  crystallographic information; 3D view; checkCIF report

## Figures and Tables

**Figure 4:**
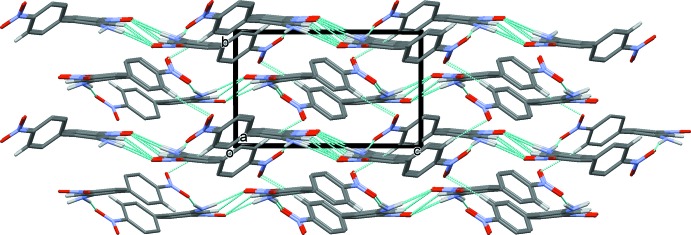
A view along the *a* axis of the crystal packing of (I). Hydrogen bonds are shown as dashed lines (see Table 1 ▸) and, for clarity, only H atoms H2*A*, H2*B*, H6 and H9 have been included.

**Table 1:** Hydrogen-bond geometry (Å, °)

*D*—H⋯*A*	*D*—H	H⋯*A*	*D*⋯*A*	*D*—H⋯*A*
N2—H2*B*⋯O1^i^	0.92 (2)	2.36 (2)	3.229 (3)	157 (2)
N2—H2*A*⋯O4^ii^	0.89 (2)	2.50 (2)	3.345 (3)	157 (2)
C6—H6⋯O1^i^	0.93	2.54	3.308 (3)	140
C9—H9⋯O3^iii^	0.93	2.57	3.496 (3)	174

Symmetry codes: (i) 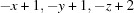; (ii) 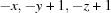; (iii) 
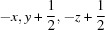
.

**Table 2:** Experimental details

Crystal data	
Chemical formula	C_12_H_9_N_3_O_4_
*M*_r_	259.22
Crystal system, space group	Monoclinic, *P*2_1_/*c*
Temperature (K)	296
*a*, *b*, *c* (Å)	14.2940 (11), 7.0352 (6), 11.6043 (9)
β (°)	99.437 (6)
*V* (Å^3^)	1151.15 (16)
*Z*	4
Radiation type	Mo *K*α
μ (mm^−1^)	0.12
Crystal size (mm)	0.34 × 0.20 × 0.07

Data collection	
Diffractometer	Stoe IPDS 2
Absorption correction	Integration (*X-RED32*; Stoe & Cie, 2002 ▸)
*T*_min_, *T*_max_	0.980, 0.993
No. of measured, independent and observed [*I* > 2σ(*I*)] reflections	6476, 2566, 1052
*R*_int_	0.044
(sin θ/λ)_max_ (Å^−1^)	0.646

Refinement	
*R*[*F* ^2^ > 2σ(*F* ^2^)], *wR*(*F* ^2^), *S*	0.040, 0.092, 0.81
No. of reflections	2566
No. of parameters	180
No. of restraints	2
H-atom treatment	H atoms treated by a mixture of independent and constrained refinement
Δρ_max_, Δρ_min_ (e Å^−3^)	0.10, −0.12

Computer programs: *X-AREA* and *X-RED32* (Stoe & Cie, 2002 ▸), *SHELXT* (Sheldrick 2015*a* ▸), *SHELXL2016* (Sheldrick, 2015*b* ▸), *ORTEP-3 for Windows* and *WinGX* (Farrugia, 2012 ▸), *Mercury* (Macrae *et al.*, 2008 ▸) and *PLATON* (Spek, 2009 ▸).

## supplementary crystallographic information

### Crystal data

**Table:**

C_12_H_9_N_3_O_4_	*F*(000) = 536
*M**_r_* = 259.22	*D*_x_ = 1.496 Mg m^−^^3^
Monoclinic, *P*2_1_/*c*	Mo *K*α radiation, λ = 0.71073 Å
*a* = 14.2940 (11) Å	Cell parameters from 3570 reflections
*b* = 7.0352 (6) Å	θ = 2.1–27.8°
*c* = 11.6043 (9) Å	µ = 0.12 mm^−^^1^
β = 99.437 (6)°	*T* = 296 K
*V* = 1151.15 (16) Å^3^	Prism, orange
*Z* = 4	0.34 × 0.20 × 0.07 mm

### Data collection

**Table:**

Stoe IPDS 2 diffractometer	2566 independent reflections
Radiation source: sealed X-ray tube, 12 x 0.4 mm long-fine focus	1052 reflections with *I* > 2σ(*I*)
Plane graphite monochromator	*R*_int_ = 0.044
Detector resolution: 6.67 pixels mm^-1^	θ_max_ = 27.4°, θ_min_ = 2.9°
rotation method scans	*h* = −18→18
Absorption correction: integration (X-RED32; Stoe & Cie, 2002)	*k* = −8→9
*T*_min_ = 0.980, *T*_max_ = 0.993	*l* = −14→11
6476 measured reflections	

### Refinement

**Table:**

Refinement on *F*^2^	Primary atom site location: structure-invariant direct methods
Least-squares matrix: full	Secondary atom site location: difference Fourier map
*R*[*F*^2^ > 2σ(*F*^2^)] = 0.040	Hydrogen site location: mixed
*wR*(*F*^2^) = 0.092	H atoms treated by a mixture of independent and constrained refinement
*S* = 0.81	*w* = 1/[σ^2^(*F*_o_^2^) + (0.0353*P*)^2^] where *P* = (*F*_o_^2^ + 2*F*_c_^2^)/3
2566 reflections	(Δ/σ)_max_ < 0.001
180 parameters	Δρ_max_ = 0.10 e Å^−^^3^
2 restraints	Δρ_min_ = −0.12 e Å^−^^3^

### Special details

**Table:**

Geometry. All esds (except the esd in the dihedral angle between two l.s. planes) are estimated using the full covariance matrix. The cell esds are taken into account individually in the estimation of esds in distances, angles and torsion angles; correlations between esds in cell parameters are only used when they are defined by crystal symmetry. An approximate (isotropic) treatment of cell esds is used for estimating esds involving l.s. planes.

### Fractional atomic coordinates and isotropic or equivalent isotropic displacement parameters (Å^2^)

**Table:**

	*x*	*y*	*z*	*U*_iso_*/*U*_eq_	
O1	0.61664 (11)	0.3867 (2)	0.93876 (16)	0.0867 (5)	
N1	0.60769 (13)	0.3912 (3)	0.8317 (2)	0.0701 (5)	
O3	−0.08732 (12)	0.2401 (3)	0.36959 (17)	0.1108 (7)	
O2	0.67457 (11)	0.3941 (3)	0.77943 (16)	0.1004 (6)	
N2	0.26998 (16)	0.4451 (3)	0.8191 (2)	0.0831 (6)	
N3	−0.02768 (15)	0.3445 (4)	0.3418 (2)	0.0899 (7)	
O4	−0.04065 (12)	0.4408 (3)	0.25269 (19)	0.1165 (7)	
C5	0.34478 (14)	0.4181 (3)	0.76003 (19)	0.0575 (5)	
C6	0.43668 (14)	0.4150 (3)	0.82181 (19)	0.0597 (5)	
H6	0.447162	0.429120	0.902589	0.072*	
C4	0.33093 (14)	0.3924 (3)	0.63808 (18)	0.0578 (5)	
C2	0.50098 (15)	0.3718 (3)	0.64471 (19)	0.0630 (6)	
H2	0.553081	0.358487	0.606754	0.076*	
C8	0.21562 (15)	0.4992 (3)	0.4654 (2)	0.0704 (6)	
H8	0.260703	0.585579	0.448436	0.084*	
C7	0.23591 (14)	0.3858 (3)	0.56438 (18)	0.0604 (5)	
C1	0.51178 (14)	0.3912 (3)	0.76354 (19)	0.0565 (5)	
C12	0.16757 (15)	0.2578 (3)	0.5889 (2)	0.0737 (6)	
H12	0.180146	0.181271	0.655006	0.088*	
C3	0.41015 (15)	0.3728 (3)	0.58389 (19)	0.0642 (6)	
H3	0.401257	0.359785	0.503076	0.077*	
C11	0.08146 (16)	0.2435 (3)	0.5161 (2)	0.0787 (7)	
H11	0.035605	0.158226	0.532243	0.094*	
C10	0.06496 (16)	0.3583 (4)	0.4192 (2)	0.0709 (6)	
C9	0.13043 (16)	0.4857 (3)	0.3926 (2)	0.0754 (6)	
H9	0.117272	0.561654	0.326283	0.090*	
H2B	0.2854 (15)	0.490 (3)	0.8945 (17)	0.106 (9)*	
H2A	0.2140 (13)	0.484 (4)	0.780 (2)	0.114 (10)*	

### Atomic displacement parameters (Å^2^)

**Table:**

	*U*^11^	*U*^22^	*U*^33^	*U*^12^	*U*^13^	*U*^23^
O1	0.0876 (11)	0.1025 (13)	0.0647 (12)	0.0106 (9)	−0.0035 (9)	−0.0071 (11)
N1	0.0702 (12)	0.0638 (12)	0.0744 (15)	0.0050 (10)	0.0068 (12)	−0.0060 (12)
O3	0.0786 (11)	0.1494 (18)	0.1005 (16)	−0.0237 (12)	0.0033 (10)	−0.0310 (13)
O2	0.0715 (10)	0.1330 (15)	0.0988 (14)	−0.0058 (11)	0.0202 (10)	−0.0176 (12)
N2	0.0719 (13)	0.1179 (18)	0.0591 (14)	−0.0019 (12)	0.0092 (11)	−0.0076 (13)
N3	0.0814 (16)	0.1079 (19)	0.0760 (18)	0.0001 (13)	0.0000 (14)	−0.0284 (14)
O4	0.1077 (14)	0.1460 (18)	0.0828 (15)	0.0023 (12)	−0.0234 (11)	0.0033 (14)
C5	0.0680 (13)	0.0557 (13)	0.0501 (13)	−0.0050 (10)	0.0135 (11)	−0.0001 (10)
C6	0.0709 (13)	0.0582 (13)	0.0481 (12)	−0.0029 (10)	0.0045 (11)	0.0007 (10)
C4	0.0708 (13)	0.0515 (12)	0.0512 (13)	−0.0050 (10)	0.0102 (11)	0.0016 (11)
C2	0.0746 (14)	0.0591 (14)	0.0573 (15)	−0.0005 (11)	0.0171 (12)	−0.0026 (11)
C8	0.0774 (14)	0.0767 (15)	0.0542 (14)	−0.0036 (12)	0.0025 (12)	0.0066 (12)
C7	0.0715 (13)	0.0594 (13)	0.0490 (13)	−0.0049 (11)	0.0063 (11)	−0.0043 (11)
C1	0.0645 (12)	0.0457 (12)	0.0580 (15)	0.0001 (10)	0.0063 (11)	−0.0007 (11)
C12	0.0873 (15)	0.0735 (15)	0.0578 (15)	−0.0149 (13)	0.0047 (13)	0.0043 (13)
C3	0.0862 (15)	0.0601 (13)	0.0468 (13)	−0.0011 (11)	0.0123 (12)	0.0004 (11)
C11	0.0838 (16)	0.0794 (16)	0.0702 (18)	−0.0193 (13)	0.0045 (13)	−0.0057 (15)
C10	0.0702 (14)	0.0817 (17)	0.0562 (16)	0.0010 (12)	−0.0033 (12)	−0.0183 (13)
C9	0.0838 (15)	0.0808 (17)	0.0577 (15)	0.0032 (13)	0.0001 (13)	0.0054 (13)

### Geometric parameters (Å, º)

**Table:**

O1—N1	1.228 (2)	C2—C1	1.369 (3)
N1—O2	1.214 (2)	C2—C3	1.372 (3)
N1—C1	1.466 (3)	C2—H2	0.9300
O3—N3	1.209 (2)	C8—C9	1.367 (3)
N2—C5	1.375 (3)	C8—C7	1.389 (3)
N2—H2B	0.922 (17)	C8—H8	0.9300
N2—H2A	0.894 (17)	C7—C12	1.392 (3)
N3—O4	1.224 (3)	C12—C11	1.378 (3)
N3—C10	1.477 (3)	C12—H12	0.9300
C5—C6	1.390 (3)	C3—H3	0.9300
C5—C4	1.408 (3)	C11—C10	1.373 (3)
C6—C1	1.370 (3)	C11—H11	0.9300
C6—H6	0.9300	C10—C9	1.368 (3)
C4—C3	1.389 (3)	C9—H9	0.9300
C4—C7	1.482 (3)		
			
O2—N1—O1	123.1 (2)	C7—C8—H8	119.5
O2—N1—C1	118.3 (2)	C8—C7—C12	118.8 (2)
O1—N1—C1	118.63 (19)	C8—C7—C4	120.41 (19)
C5—N2—H2B	115.9 (14)	C12—C7—C4	120.6 (2)
C5—N2—H2A	119.6 (17)	C2—C1—C6	122.8 (2)
H2B—N2—H2A	116 (2)	C2—C1—N1	119.0 (2)
O3—N3—O4	123.1 (2)	C6—C1—N1	118.2 (2)
O3—N3—C10	118.6 (3)	C11—C12—C7	120.5 (2)
O4—N3—C10	118.3 (3)	C11—C12—H12	119.7
N2—C5—C6	119.4 (2)	C7—C12—H12	119.7
N2—C5—C4	121.8 (2)	C2—C3—C4	122.7 (2)
C6—C5—C4	118.80 (19)	C2—C3—H3	118.6
C1—C6—C5	119.9 (2)	C4—C3—H3	118.6
C1—C6—H6	120.1	C10—C11—C12	118.4 (2)
C5—C6—H6	120.1	C10—C11—H11	120.8
C3—C4—C5	118.49 (19)	C12—C11—H11	120.8
C3—C4—C7	118.3 (2)	C9—C10—C11	122.6 (2)
C5—C4—C7	123.25 (18)	C9—C10—N3	119.0 (3)
C1—C2—C3	117.28 (19)	C11—C10—N3	118.4 (2)
C1—C2—H2	121.4	C8—C9—C10	118.6 (2)
C3—C2—H2	121.4	C8—C9—H9	120.7
C9—C8—C7	121.0 (2)	C10—C9—H9	120.7
C9—C8—H8	119.5		
			
N2—C5—C6—C1	−179.0 (2)	O2—N1—C1—C6	−170.96 (19)
C4—C5—C6—C1	1.3 (3)	O1—N1—C1—C6	9.7 (3)
N2—C5—C4—C3	177.7 (2)	C8—C7—C12—C11	0.2 (3)
C6—C5—C4—C3	−2.6 (3)	C4—C7—C12—C11	−175.9 (2)
N2—C5—C4—C7	−2.1 (3)	C1—C2—C3—C4	0.0 (3)
C6—C5—C4—C7	177.60 (19)	C5—C4—C3—C2	2.0 (3)
C9—C8—C7—C12	−0.3 (3)	C7—C4—C3—C2	−178.2 (2)
C9—C8—C7—C4	175.8 (2)	C7—C12—C11—C10	0.0 (3)
C3—C4—C7—C8	−50.6 (3)	C12—C11—C10—C9	−0.1 (3)
C5—C4—C7—C8	129.1 (2)	C12—C11—C10—N3	−179.4 (2)
C3—C4—C7—C12	125.4 (2)	O3—N3—C10—C9	−175.5 (2)
C5—C4—C7—C12	−54.9 (3)	O4—N3—C10—C9	3.9 (3)
C3—C2—C1—C6	−1.4 (3)	O3—N3—C10—C11	3.8 (3)
C3—C2—C1—N1	−179.90 (18)	O4—N3—C10—C11	−176.8 (2)
C5—C6—C1—C2	0.7 (3)	C7—C8—C9—C10	0.2 (3)
C5—C6—C1—N1	179.24 (19)	C11—C10—C9—C8	0.0 (3)
O2—N1—C1—C2	7.6 (3)	N3—C10—C9—C8	179.3 (2)
O1—N1—C1—C2	−171.76 (19)		

### Hydrogen-bond geometry (Å, º)

**Table:**

*D*—H···*A*	*D*—H	H···*A*	*D*···*A*	*D*—H···*A*
N2—H2*B*···O1^i^	0.92 (2)	2.36 (2)	3.229 (3)	157 (2)
N2—H2*A*···O4^ii^	0.89 (2)	2.50 (2)	3.345 (3)	157 (2)
C6—H6···O1^i^	0.93	2.54	3.308 (3)	140
C9—H9···O3^iii^	0.93	2.57	3.496 (3)	174

Symmetry codes: (i) −*x*+1, −*y*+1, −*z*+2; (ii) −*x*, −*y*+1, −*z*+1; (iii) −*x*, *y*+1/2, −*z*+1/2.
